# The method of solution of equations with coefficients that contain measurement errors, using artificial neural network

**DOI:** 10.1007/s00521-012-1239-0

**Published:** 2012-11-02

**Authors:** Konrad Zajkowski

**Affiliations:** Division of Electrotechnics and Electronics, Technical University of Koszalin, 15-17 Raclawicka St., 75-620 Koszalin, Poland

**Keywords:** Artificial neural network, Measurement errors, Induction motor model, Parameter identification

## Abstract

This paper presents an algorithm for solving *N*-equations of *N*-unknowns. This algorithm allows to determine the solution in a situation where coefficients *A*_*i*_ in equations are burdened with measurement errors. For some values of *A*_*i*_ (where *i* = 1,…, *N*), there is no inverse function of input equations. In this case, it is impossible to determine the solution of equations of classical methods.

## Introduction

Mathematical models that describe electric dependencies in the receiver tested are built from discrete components. For a full description of such a model, it is required to identify the parameters *x*_*i*_ of these elements (see Eq. 1). Most frequently, this identification is carried out indirectly through the measurements of electrical quantities *A*_*i*_ on the object tested [5, 8]. The parameters sought are determined from the mathematical relations (1) that describe the object.

1 $$  \left\{ {\begin{array}{*{20}c}    {A_{1}  = f_{1} \left( {y_{1} ,y_{2} , \ldots ,y_{i} , \ldots ,y_{N} } \right)} \hfill  \\    {A_{2}  = f_{2} \left( {y_{1} ,y_{2} , \ldots ,y_{i} , \ldots ,y_{N} } \right)} \hfill  \\     \vdots  \hfill  \\    {A_{i}  = f_{i} \left( {y_{1} ,y_{2} , \ldots ,y_{i} , \ldots ,y_{N} } \right)} \hfill  \\     \vdots  \hfill  \\    {A_{N}  = f_{N} \left( {y_{1} ,y_{2} , \ldots ,y_{i} , \ldots ,y_{N} } \right)} \hfill  \\   \end{array} } \right. $$

where *f*_*i*_ certain functions depending on the model, *A*_*i*_ the values measured, *y*_*i*_ parameters that describe the model.

The classic method to solve Eq. (1) consists in determining inverse functions (2). Measurement inaccuracies that are contained in *A*_*i*_ are transferred to parameters *y*_*i*_ to be determined.

2 $$ \left\{ {\begin{array}{*{20}c}    {y_{1}  = g_{1} \left( {A_{1} ,A_{2} , \ldots ,A_{N} } \right)} \hfill  \\    {y_{2}  = g_{2} \left( {A_{1} ,A_{2} , \ldots ,A_{N} } \right)} \hfill  \\     \vdots  \hfill  \\    {y_{N}  = g_{N} \left( {A_{1} ,A_{2} , \ldots ,A_{N} } \right)} \hfill  \\   \end{array} } \right. $$

In some cases, the determination of Eq. (2) may not be possible [14, 16]. This means that for adopted coefficients *A*_*i*_, there are no inverse functions *g*_*i*_. Eq. (2) are determined for the values of environment *A*_*i*_ and which contain measurement errors. In this case, approximate solutions are sought which satisfy Relation (3).

3 $$ \left| {A_{i} - f_{i} \left( {y_{1} ,y_{2} , \ldots ,y_{N} } \right)} \right| \approx 0 $$

The solution will be close to coefficients *A*_*i*_.

## An example of a model for identification

**Fig. 1:**
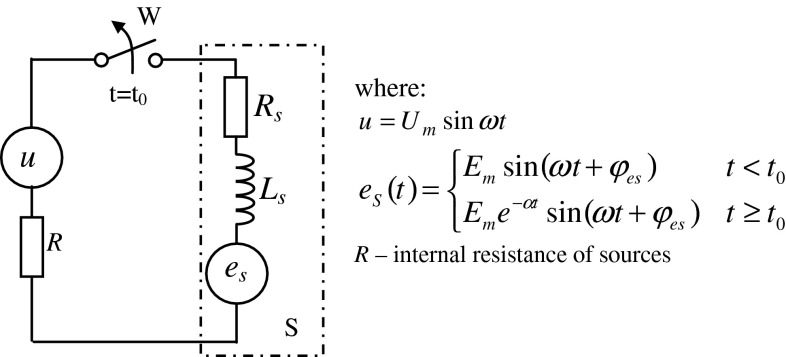
Electric model of the motor and the power source, where $$ u = U_{m} \sin \omega t,\quad e_{S} (t) = \left\{ {\begin{array}{*{20}c} {E_{m} \sin (\omega t + \phi_{es} )} \hfill & {t < t_{0} } \hfill \\ {E_{m} e^{ - \alpha t} \sin (\omega t + \phi_{es} )} \hfill & {t \ge t_{0} } \hfill \\ \end{array} } \right., $$ *R* internal resistance of sources

The analysis covered a single phase on an induction motor. The purpose of the analysis is to determine current–voltage dependences on the terminals of one motor phase. These relationships can be determined from the model that consists of serially connected elements: *R*_*s*_, *L*_*s*_ i *e*_*s*_ (Fig. 1).

Coefficients *R*_*S*_, *L*_*S*_, *E*_*m*_, φ_*es*_, *α* that are being sought represent many of the phenomena that occur in the motor and the system that is driven. For example, the inertia of the rotor and the system driven will affect *e*_*s*_, and the angular velocity will exert an influence on mutual inductances, which are described with *L*_*S*_. When searching for the parameters of the model, the fact is also important that these factors cannot be determined with the engine being stopped. This means that the *R*_*S*_ does not reflect the winding resistance and *L*_*S*_ does not reflect their inductance. The parameters of the model are defined for a constant load on the machine shaft and for constant rotations. When changing the load, the parameters of the model change, as well.

In this situation, the parameters that are being determined cannot in any way be unified. They should be determined for a specific drive train (the motor and the machine driven). These parameters can vary considerably for the same engine with different mechanical properties of the system driven.

**Fig. 2:**
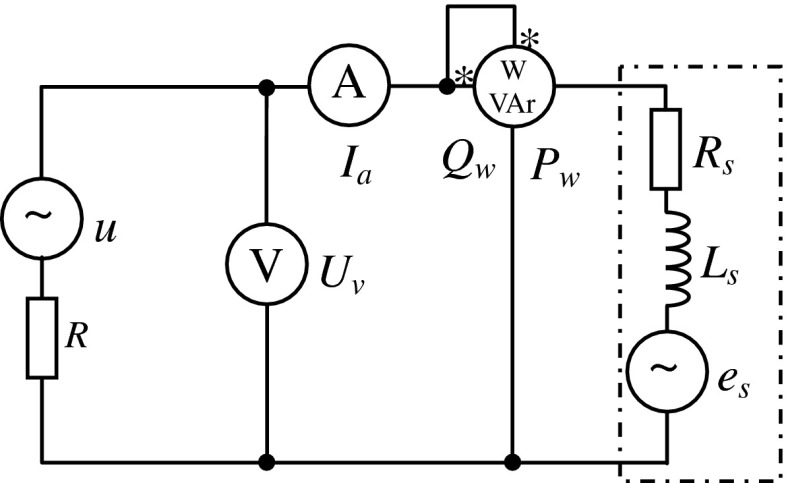
Measuring circuit for parametric identification

The identification of the model consists in searching for *E*_*m*_, φ_*es*_, *R*_*S*_, and *L*_*S*_. These parameters can be determined on the receiver [6, 7, 9–11] by making measurements in the steady state (in the case of an induction motor: during operation with a constant load and a constant speed) in the system as shown below (Fig. 2):

According to the model adopted, we know that:

4 $$ e_{s} = E_{s} \sqrt 2 \sin \left( {\omega t + \phi_{es} } \right). $$

In the field of complex numbers, the following can be written:

5 $$ \underline{E}_{s} = E_{s} e^{{j\phi_{es} }} , $$

6 $$ \underline{U} = Ue^{{j\phi_{u} }} . $$

For one mesh, the voltage equation is as follows:

7 $$ \underline{I}_{a} \left( {R_{s} + R + jX_{s} } \right) + \underline{E}_{s} - \underline{U} = 0, $$

where $$ X_{S} = \omega L_{S} . $$

Next, by transforming (7), we determine current *I*_*a*_:

8 $$ \underline{I}_{a} = \frac{{\underline{U} - \underline{E}_{s} }}{{R_{s} + R + jX_{s} }} $$

Voltmeter *V* measures the difference in the supply voltage and in the voltage drop across internal resistance *R*. Thus, in the field of complex numbers, there will be the following:

9 $$ \underline{U}_{V} = \underline{U} - \underline{I}_{a} R. $$

From Eqs. (8) and (9), one can obtain the following:

10 $$ \underline{U}_{V} = \frac{1}{{R_{s} + R + jX_{s} }}\left[ {\underline{U} \left( {R_{s} + jX_{s} } \right) + \underline{E}_{s} R} \right]. $$

Knowing that the forces and the current are equal, respectively:

$$ P_{w} = \text{Re} \left( {\underline{U}_{v} \underline{I}_{a}^{*} } \right),\quad Q_{w} = \text{Im} \left( {\underline{U}_{v} \underline{I}_{a}^{*} } \right),\quad \underline{I}_{a}^{*} = \frac{{\underline{U}^{*} - \underline{E}_{s}^{*} }}{{R_{s} + R - jX_{s} }} $$

We obtain the following equations:

11 $$ \left\{ {\begin{array}{*{20}c} {I_{a} = \left| {\frac{{\underline{U} - \underline{E}_{s} }}{{R_{s} + R + jX_{s} }}} \right|} \hfill \\ {U_{v} = \left| {\frac{1}{{R_{s} + R + jX_{s} }}\left[ {\underline{U} (R_{s} + jX_{s} ) + \underline{E}_{s} R} \right]} \right|} \hfill \\ {P_{w} = \text{Re} \left\{ {\frac{{\left[ {\underline{U} (R_{s} + jX_{s} ) + \underline{E}_{s} R} \right] \cdot \left[ {\underline{U}^{*} - \underline{E}_{s}^{*} } \right]}}{{(R_{s} + R)^{2} + X_{s}^{2} }}} \right\}} \hfill \\ {Q_{w} = \text{Im} \left\{ {\frac{{\left[ {\underline{U} (R_{s} + jX_{s} ) + \underline{E}_{s} R} \right] \cdot \left[ {\underline{U}^{*} - \underline{E}_{s}^{*} } \right]}}{{(R_{s} + R)^{2} + X_{s}^{2} }}} \right\}} \hfill \\ \end{array} } \right.. $$

Equation (11) is consistent with (1). The coefficients of the model of the receiver that are obtained from the above equations are not determinable for all the input parameters (*U*_*V*_, *I*_*a*_, *P*_*W*_, *Q*_*W*_). There are those areas that result from measurement inaccuracies where the system of Eq. (11) has no solutions.

It was found that these coefficients cannot be determined using the Newton’s interpolation algorithm [15, 16]. There are no functions that are inverse to Eq. (11), either.

Process time constant 1/α that is being sought, and which is mainly related to the inertia of the rotor and the system driven, can be determined experimentally by observing the course of voltage versus time at the motor terminals immediately after commutation.

In [13], the authors proved that amplitude *E*_*S*_ can be equal to amplitude *U*. In this paper, it was also observed that frequency *E*_*S*_ is similar to the frequency of the mains voltage. It was also noted that phase shift φ_*es*_ is equal to 0.

In this model, it is assumed that the frequencies of both sources are identical. This assumption does not substantially affect the results of further simulations.

## Construction of an artificial neural network

Coefficients *E*_*m*_, *R*_*S*_, and *L*_*S*_ can be determined from Eq. (11) using a neural network. The network input parameters *x*_1_ = *U*_*v*_ [V], *x*_2_ = *I*_*a*_ [A], *x*_3_ = *P*_*w*_ [W], *x*_4_ = *Q*_*w*_ [VAr] contain measurement errors. Due to the nature of the adopted activation function [1–3], the output neuron of the output layer must be within range $y \in \left( {0,\,1} \right)$. The initial values were as follows: *y*_1_ = *R*_*s*_ [kΩ], *y*_2_ = *L*_*s*_ [H], *y*_3_ = *E*_*s*_ [kV].

Training of the network must be for those learning vectors $\sigma = \left[ {x_{1} , \ldots ,x_{{N_{0} }} ,\left| {,d_{1}^{\left( L \right)} , \ldots ,d_{{N_{L} }}^{\left( L \right)} } \right.} \right]$ that do not contain any measurement errors. Learning vectors are constructed from Eqs. (1) or (11) for random values *y*_1_, *y*_2_, *y*_3_ that lie within the set of permissible changes, and which is limited with values *a* and *b* [4, 12].

**Fig. 3:**
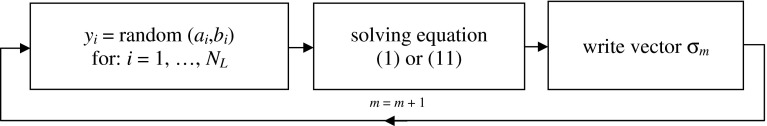
Construction of learning vectors

The test vector is built according to Fig. 3 for values *y*_*i*_ that are not contained within the training set.

The neural network was built in a VBA environment in EXCEL.

**Fig. 4:**
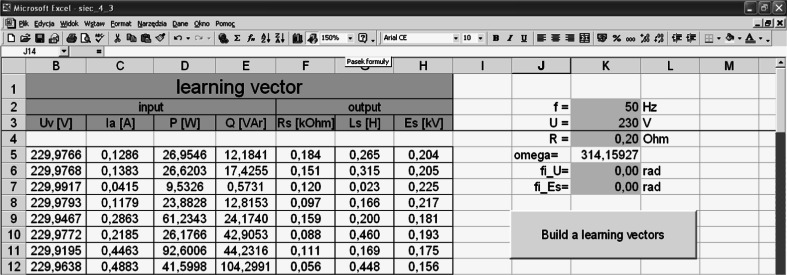
The workbook that builds learning vectors



The script associated with the button in Fig. 4 determines the random values: $d_{1} = R_{s} \in \left\langle {0.005 \div 0.2} \right\rangle {\text{k}}\Upomega ,\quad d_{2} = L_{s} \in \left\langle {0.005 \div 0.5} \right\rangle {\text{H}},\quad d_{3} = E_{s} \in \left\langle {0.15 \div U} \right\rangle {\text{kV}}$.

Further values *U*_*v*_, *I*_*a*_, *P*_*w*_ and *Q*_*w*_ are determined from Eq. (11).

**Fig. 5:**
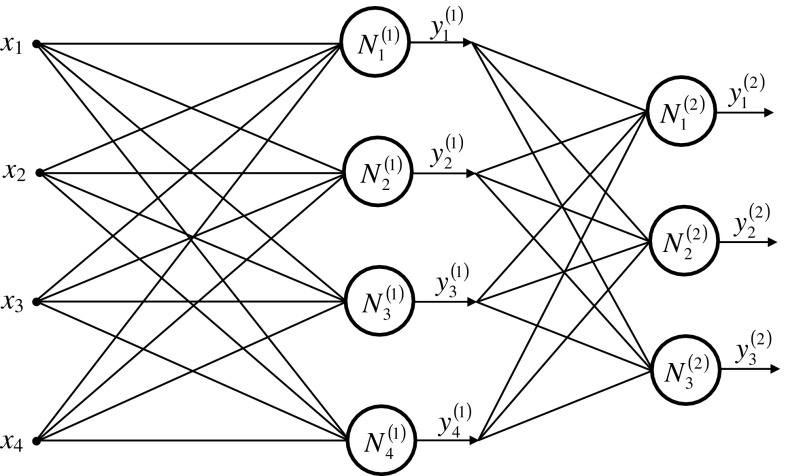
Structure of the neural network

After tests of several neural networks, a decision was made to build a neural network with topology (Fig. 5), with one hidden layer. The weights of neurons are determined by back propagation.

**Fig. 6:**
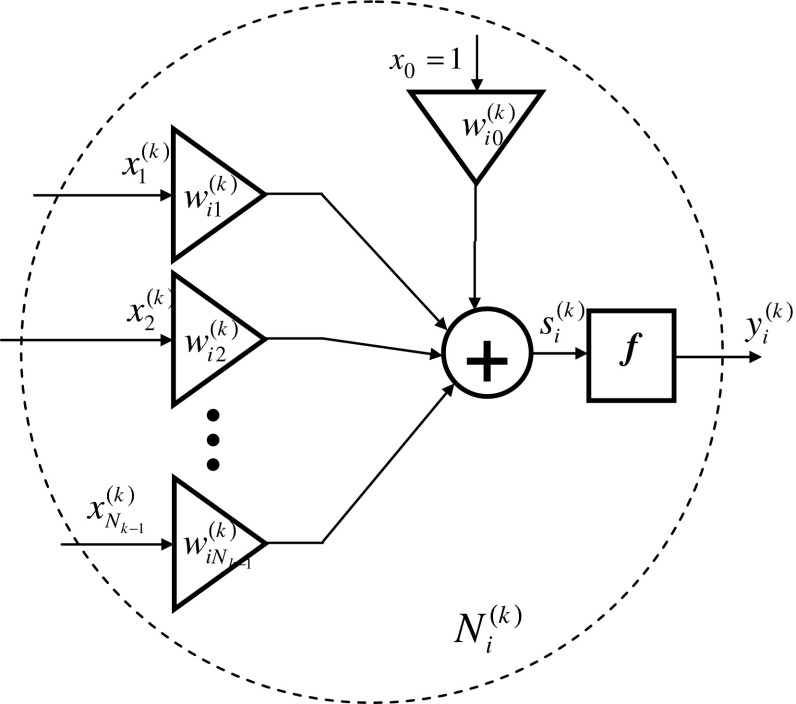
Diagram of neuron $N_{i}^{(k)}$

Individual neurons in the network are structured according to Fig. 6.

In the network being built, the following indications were accepted:

*t*: iteration step, *t* = 1, 2, …
$$ y_{i}^{(k)} (t) $$
*i*th output of the neuron $N_{i}^{(k)}$
$$ y_{i}^{\left( L \right)} \left( t \right) $$
*i*th output of the network
*k*: network layer, *k* = 1, …, *L*
*L*: network output layer, the number of network layers
*i*: neuron number in layer, *i* = 1,…, *N*_*k*_
$$ x_{j}^{(k)} (t) $$
input signal in the *k*th layer
$$ x_{j} (t) = x_{j}^{(1)} (t) $$
input of the network
*j*: number of the input signal in the *k*th layer, *j* = 1,…, *N*_*k*−1_
*N*_0_: number of inputs to the network
*N*_*k*_: number of neurons in the *k*th layer
*N*_*L*_: number of neurons in the last layer
$$ s_{i}^{(k)} (t) $$
neuron membrane potential $N_{i}^{(k)}$ in the *k*th layer
$$ w_{i,j}^{(k)} (t) $$
weight of the *j*th input of the *i*th neuron $N_{i}^{(k)}$ in the *k*th layer
$$ d_{i}^{(L)} (t) $$
*i*th reference signal output from the learning vector
$$ \varepsilon_{i}^{(L)} (t) $$
error of the *i*th network output, $\varepsilon_{i}^{(L)} (t) = d_{i}^{(L)} (t) - y_{i}^{(L)} (t)$
η: network learning rate
*Q*(*t*): error at the output of the network for one reference vector
Q^*^(*t*): error at the output of the network for the entire epoch

The output of neuron $N_{i}^{(k)}$ (Fig. 6) at time *t* is described with the following relation:

12 $$ y_{i}^{(k)} (t) = f\left( {s_{i}^{(k)} (t)} \right) $$

while membrane potential $s_{i}^{(k)}$ is equal to:

13 $$ s_{i}^{(k)} (t) = \sum\limits_{j = 0}^{{N_{k - 1} }} {w_{i,j}^{(k)} (t) \cdot x_{j}^{(k)} (t)} $$

The input neuron for *k* = 1 layer is equal to network inputs $x_{j} (t) = x_{j}^{(1)} (t)$. Each layer has one input $x_{0}^{(k)} (t) = 1$. Other inputs are the outputs of the previous layer.

14 $$ x_{j}^{k} (t) = \left\{ {\begin{array}{*{20}c} {x_{j} (t)} \hfill & {{\text{for }}k = 1} \hfill \\ {y_{j}^{k - 1} (t)} \hfill & {{\text{for }}k = 2, \ldots ,L} \hfill \\ 1 \hfill & {{\text{for }}j = 0,\quad k = 1, \ldots ,L} \hfill \\ \end{array} } \right. $$

The error at the output of the network for one learning vector *σ* is:

15 $$ Q(t) = \sum\limits_{i = 1}^{{N_{L} }} {(\varepsilon_{i}^{L} (t))^{2} } = \sum\limits_{i = 1}^{{N_{L} }} {(d_{i}^{L} (t) - y_{i}^{L} (t))^{2} } $$

The weights of the individual neuron inputs are determined from the steepest descent rule:

16 $$ w(t + 1) = w(t) - \eta \cdot {\mathbf{g}}(w(t)) $$

where $$ {\mathbf{g}}(w(t)) = \left[ {\begin{array}{*{20}c} {\frac{\partial Q\left( t \right)}{{\partial w_{1} \left( t \right)}},} & {\frac{\partial Q\left( t \right)}{{\partial w_{2} \left( t \right)}},} & {\frac{\partial Q\left( t \right)}{{\partial w_{3} \left( t \right)}},} & { \ldots ,} & {\frac{\partial Q\left( t \right)}{{\partial w_{n} \left( t \right)}}} \\ \end{array} } \right]^{T} $$ is the vector gradient.

From Eq. (16), for any weight in any layer, the following is obtained:

17 $$ w_{i,j}^{(k)} (t + 1) = w_{i,j}^{(k)} (t) - \eta \frac{\partial Q(t)}{{\partial w_{i,j}^{(k)} (t)}} = w_{i,j}^{(k)} (t) + 2\eta \delta_{i}^{(k)} (t)x_{j}^{(k)} (t) $$

Parameter $\delta_{i}^{(k)} (t)$ is determined differently than for the output layer and the hidden layer:

18 $$ \delta_{i}^{(k)} (t) = \left\{ {\begin{array}{*{20}c} {\varepsilon_{i}^{(L)} (t) \cdot f^{'} \left( {s_{i}^{(L)} (t)} \right)} \hfill & {{\text{for }}k = L} \hfill \\ {f^{'} \left( {s_{i}^{(k)} (t)} \right) \cdot \sum\limits_{m = 1}^{{N_{k + 1} }} {\delta_{m}^{(k + 1)} (t)w_{i,m}^{(k + 1)} (t)} } \hfill & {{\text{for }}k \ne L} \hfill \\ \end{array} } \right. $$

where $$ \varepsilon_{i}^{(L)} (t) = d_{i}^{(L)} (t) - y_{i}^{(L)} (t). $$

Network training is carried out by an incremental updating of weights, that is, each time after the entry of a successive learning vector, responses are determined and the weights are modified. The simulation is continued until the total output error for entire epoch *Q* ^*^(*t*) is smaller than the accepted set *Q*_min_.

19 $$ Q^{*} (t) = \sum\limits_{m = 1}^{M} {Q_{m} (t) \le Q_{ \hbox{Min} } } $$

where *M* is the number of learning vectors in the epoch.

The neuron activation function was adopted as a continuous unipolar function of the signum type:

20 $$ f(s_{i} (t)) = \frac{1}{{1 + e^{{ - \beta \cdot s_{i} (t)}} }} $$

where *β* is the steepness factor.

With low values of coefficient *β*, the function is usually mild. By increasing *β*, the plot becomes steeper until the threshold course is obtained.

The derivative of the activation function is as follows:

21 $$ f^{'} (s_{i} (t)) = \frac{{\beta \cdot e^{{ - \beta \cdot s_{i} (t)}} }}{{\left( {1 + e^{{ - \beta \cdot s_{i} (t)}} } \right)^{2} }} = \beta \cdot f(s_{i} (t)) \cdot (1 - f(s_{i} (t))) $$

**Fig. 7:**
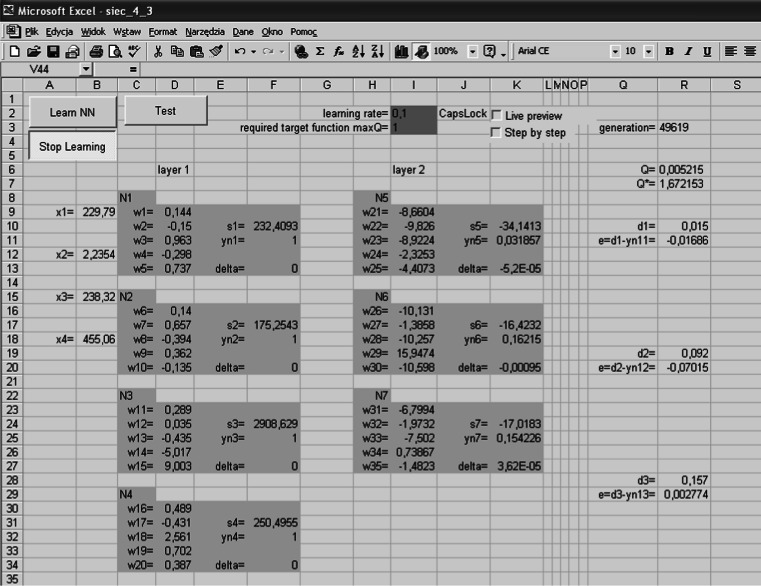
Sheet for the visualization of the network operation

**Fig. 8:**
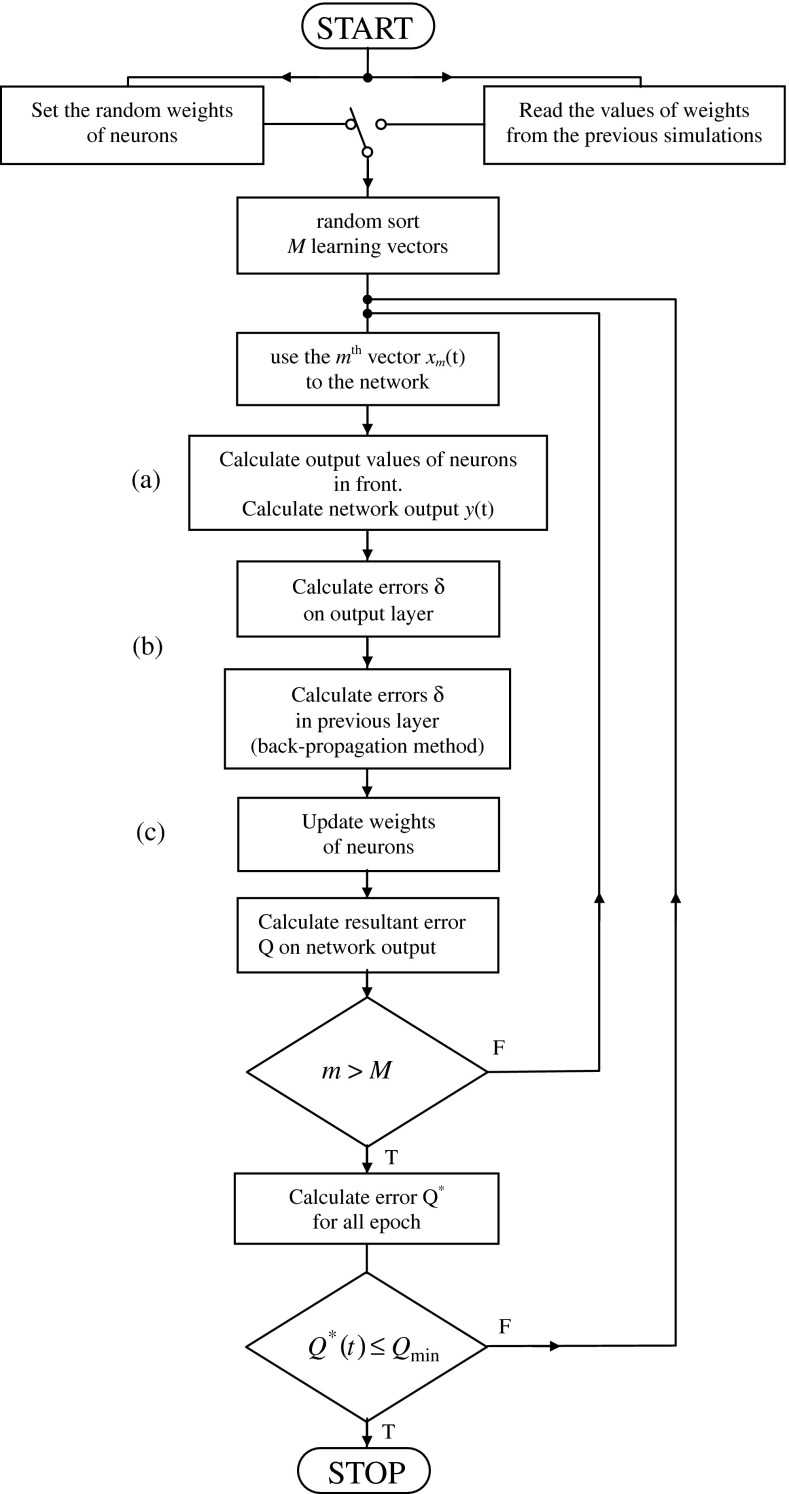
Block diagram of the network learning algorithm

The calculation sheet in Fig. 7 allows an observation of the characteristic values of the network tested. Starting of the network training produces a script written in VBA that executes in a loop of a neural network algorithm according to (12) ÷ (21) and the block diagram in Fig. 8.

**Fig. 9:**
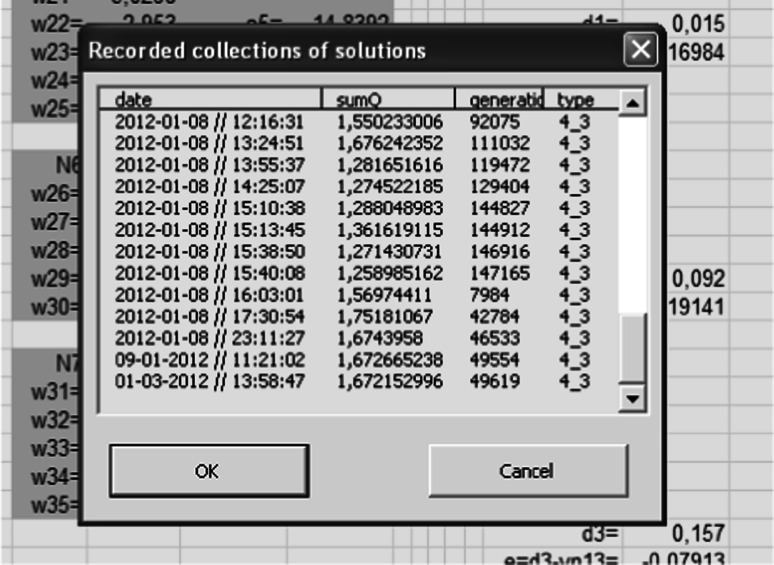
The reading window of the recorded data

The start of the algorithm is possible for the weights that are selected at random from range $\left\langle { - 1,\,1} \right\rangle$ or the reading stored from the previous simulations (Fig. 9).

## Learning of the network

The set of learning vectors that form one epoch consists of 200 elements. Owing to the ability to read and write data, it is possible to pause the simulation and to change its parameters during operation [4, 12].

**Fig. 10:**
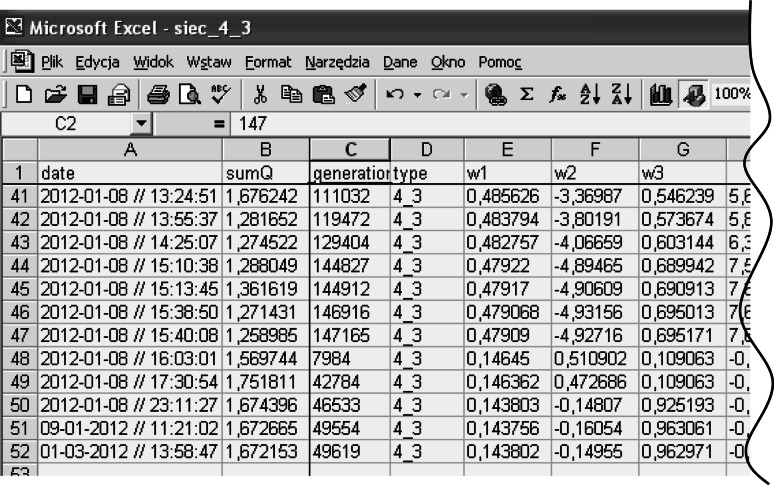
Sheet with the saved results of the simulation

Reading of the stored data allows a continuation of the previously stopped simulation. The window in Fig. 9 retrieves the values from the appropriate data sheet (Fig. 10).

The output values of the neurons (Fig. 8a) are determined by analyzing the neurons in layers starting from the input layer; the output layer comes last.



For all the layers, the steepness factor *β* (20) of activation function *fx* was assumed as equal to 0.1.

The determination of value $\delta_{i}^{(k)} (t)$ (Fig. 8b) shall be in accordance with Formula (18). This determination takes place starting from the output layer; the input layer comes last.



Correction of the values of weights (Fig. 8c) is carried out according to Relation (17).



**Fig. 11:**
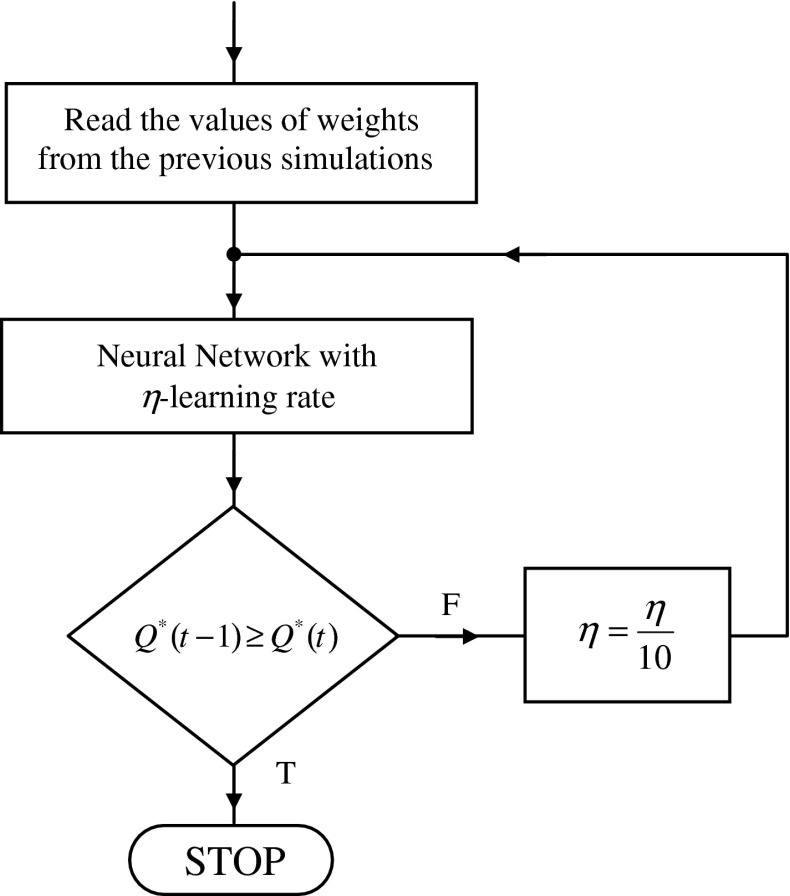
The method to reduce the coefficient of network learning

Network learning factor *η* from Formula (17) was adopted on the first stage of the simulation as being constant and equal to 0.1. After an analysis of ca. 70,000 epochs, the value of target function *Q* ^*^(*t*), which was calculated in accordance with Formula (19), began to oscillate on the level of 1.42. A decrease in *Q* ^*^(*t*) occurred only after a reduction in network learning rate *η*. The correct procedure for the network training should provide for an ability to change this ratio during the analysis (Fig. 11).

Oscillations around the optimal solution are manifested with a momentary increase in the value of *Q* ^*^(*t*).

22 $$ Q^{*} (t) \ge Q^{*} (t - 1) $$

**Fig. 12:**
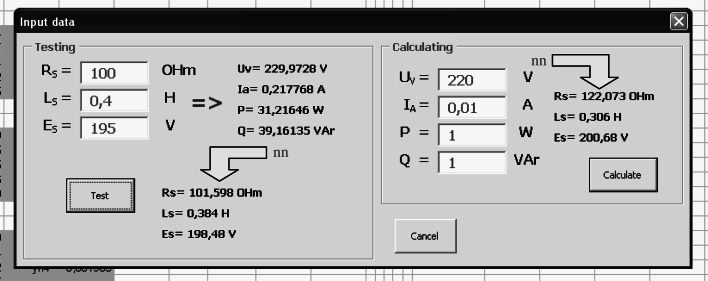
The window for testing and results analysis

Once the required value of *Q* ^*^(*t*) from Eq. (19) has been reached, the network test is performed (Fig. 12).

## Network test

The network test consists in determining the values of *U*_*V*_ *, I*_*a*_ *, P*_*W*_, and *Q*_*W*_ from Relation (11). These values are then substituted into the neural network input, whose solution is *R*_*S*_ *, L*_*S*_, and *E*_*S*_. The window in Fig. 12 also allows a determination of the network’s solution for a selected set of weights.

Table 1 illustrates the network test for randomly selected values of *R*_*S*_ *, L*_*S*_, and *E*_*S*_.

**Table 1:** Verification of the network error

Random values			Network input				Network output			Relative error			
			*x*_1_	*x*_2_	*x*_3_	*x*_4_	*y*_1_	*y*_2_	*y*_3_	*δ*_1_	*δ*_2_	*δ*_3_	*δ*
*R*_*S*_	*L*_*S*_	*E*_*S*_	*U*_*V*_	*I*_*a*_	*P*	*Q*	*R’*_*S*_	*L’*_*S*_	*E’*_*S*_				
[Ω]	[H]	[V]	[V]	[A]	[W]	[VAr]	[Ω]	[H]	[V]	–	–	–	–
20	0.2	180	229.95	0.7576	53.21	165.88	19.721	0.210	168.626	−0.014	0.050	−0.063	0.127
151	0.315	193	229.97	0.2048	39.395	25.7897	147.083	0.354	192.849	−0.026	0.124	−0.001	0.151
150	0.09	197	229.96	0.2159	48.794	9.18694	152.107	0.093	197.019	0.014	0.033	0.0001	0.048
147	0.3	197	229.97	0.1888	36.563	23.415	147.413	0.352	192.976	0.003	0.173	−0.020	0.197
47	0.46	193	229.99	0.2434	17.368	53.211	41.373	0.409	195.946	−0.120	−0.111	0.015	0.246
150	0.09	193	229.95	0.2421	54.707	10.301	151.590	0.092	196.866	0.011	0.022	0.020	0.053
45	0.448	190	229.98	0.2706	19.015	59.256	41.182	0.408	195.771	−0.085	−0.089	0.030	0.205
145	0.4	190	229.97	0.2083	36.218	31.353	145.773	0.357	192.810	0.005	−0.108	0.015	0.128
50	0.03	160	229.73	1.3705	309.422	58.163	58.641	0.026	159.829	0.173	−0.133	−0.001	0.307
150	0.1	200	229.96	0.1955	44.0056	9.20584	152.394	0.093	197.103	0.016	−0.070	−0.014	0.100
140	0.317	189	229.96	0.2384	44.6931	31.755	146.133	0.354	192.544	0.044	0.117	0.019	0.179
40	0.317	195	229.98	0.3259	28.036	69.507	40.416	0.402	195.029	0.010	0.268	0.0002	0.279
135	0.4	195	229.97	0.1896	31.937	29.691	143.751	0.358	192.972	0.065	−0.105	−0.010	0.180
145	0.3	190	229.96	0.2311	44.568	28.936	146.443	0.353	192.636	0.010	0.177	0.014	0.201
140	0.4	190	229.97	0.2125	36.378	32.615	145.157	0.356	192.790	0.037	−0.110	0.015	0.162

The relative error for all the output neurons for the randomly adopted input vectors is:

23 $$ \delta_{1} = \frac{{y_{1} - R_{S} }}{{R_{S} }},\quad \delta_{2} = \frac{{y_{2} - L_{S} }}{{L_{S} }},\quad \delta_{3} = \frac{{y_{3} - E_{S} }}{{E_{S} }} $$

The total network error for the accepted values of *R*_*S*_ *, L*_*S*_, and *E*_*S*_ are:

24 $$ \delta = \left| {\frac{{y_{1} - R_{S} }}{{R_{S} }}} \right| + \left| {\frac{{y_{2} - L_{S} }}{{L_{S} }}} \right| + \left| {\frac{{y_{3} - E_{S} }}{{E_{S} }}} \right| . $$

**Fig. 13:**
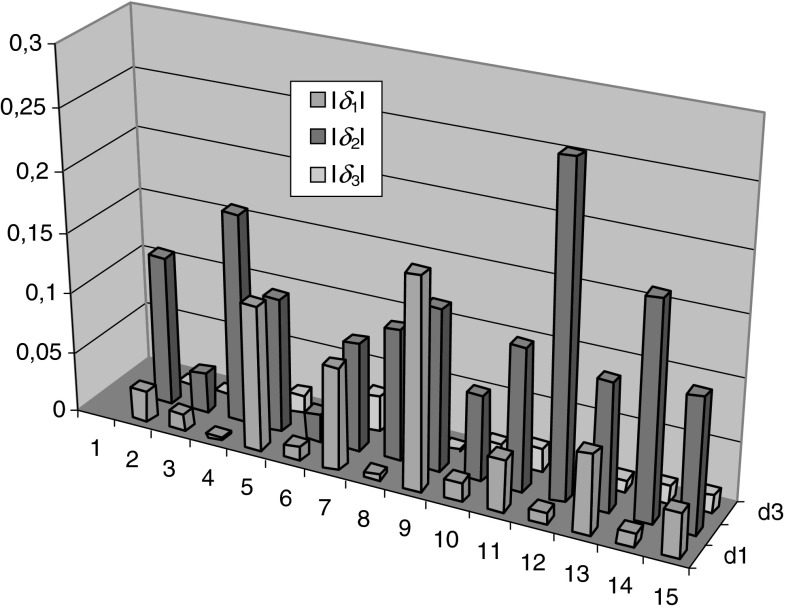
Mistake made by the network

The percentage error made by the network is determined from the largest error (the top bar in the chart in Fig. 13), and it is equal to 26.8 %.

## Conclusions

The large error value is shown for the input values that occur least frequently in the training set. An improved performance is possible by enlarging the training set or by reducing the range of acceptable changes of the values being sought.

Owing to the method presented of the selection of the electrical model parameters from the values that are measured on the receiver, it is not required to build any complex physical and electrical dependences. The engineering method of voltage, current, and power measurement allows one to determine the parameters of the model for constant electrical and mechanical conditions in the engine. The method presented is particularly useful in situations where measurement errors make it impossible to solve Eq. (2).

Building of a network with the use of the VBA environment is relatively simple. It requires the knowledge of the language basics. An important advantage of this approach is the ability to build its own networks of any topology. The design loop iteration depends largely on how one defines those variables that describe the network.

In the present solution, the individual variables occupy adjacent bytes of the memory. A sample definition of the variable holding the weights of neurons is:

$$ {\text{Public}}\;{\text{w(}}1{\text{ To Lweights)As}}\;{\text{Single}} $$

where Lweights is the number of weights of all neurons.This solution facilitates the construction of a loop program, but special attention is to be paid to assigning the weight number with the neuron number.

An alternative is to build one’s own variable (using the opportunity to build one’s own type of variables) that represents the neuron, and then group all the parameters that describe the type of the neuron in this variable. This approach will make the program more transparent, but there are problems in the construction of iterative loops. This will make the source code longer and will require more CPU load.
